# Health of Philippine Emigrants Study (HoPES): study design and rationale

**DOI:** 10.1186/s12889-018-5670-8

**Published:** 2018-06-20

**Authors:** Gilbert C. Gee, A. B. de Castro, Catherine M. Crespi, May C. Wang, Karen Llave, Eleanor Brindle, Nanette R. Lee, Maria Midea M. Kabamalan, Anna K. Hing

**Affiliations:** 1 UCLA Fielding School of Public Health, 650 Charles E. Young Drive South, Los Angeles, CA 90095-1772 USA; 2 University of Washington School of Nursing, Box 357260, Seattle, WA 98195 USA; 3 Center for Studies in Demography and Ecology, University of Washington, Box 353412, Seattle, WA 98195 USA; 4 Department of Anthropology, Sociology, and History, USC-Office of Population Studies Foundation, Inc., University of San Carlos, Talamban, Cebu City, Philippines; 5 Population Institute, College of Social Sciences and Philosophy, University of the Philippine, Diliman, Quezon City, Philippines

**Keywords:** Immigration, Obesity, Prospective, Acculturation, Asian American, Natural experiment, Stress, Ethnicity, Race

## Abstract

**Background:**

Immigrants to the United States are usually healthier than their U.S.-born counterparts, yet the health of immigrants declines with duration of stay in the U.S. This pattern is often seen for numerous health problems such as obesity, and is usually attributed to acculturation (the adoption of “American” behaviors and norms). However, an alternative explanation is secular trends, given that rates of obesity have been rising globally. Few studies of immigrants are designed to distinguish the effects of acculturation versus secular trends, in part because most studies of immigrants are cross-sectional, lack baseline data prior to migration, and do not have a comparison group of non-migrants in the country of origin. This paper describes the Health of Philippine Emigrants Study (HoPES), a study designed to address many of these limitations.

**Methods:**

HoPES is a dual-cohort, longitudinal, transnational study. The first cohort consisted of Filipinos migrating to the United States (*n* = 832). The second cohort consisted of non-migrant Filipinos who planned to remain in the Philippines (*n* = 805). Baseline data were collected from both cohorts in 2017 in the Philippines, with follow-up data collection planned over 3 years in either the U.S. for the migrant cohort or the Philippines for the non-migrant cohort. At baseline, interviewers administered semi-structured questionnaires that assessed demographic characteristics, diet, physical activity, stress, and immigration experiences. Interviewers also measured weight, height, waist and hip circumferences, blood pressure, and collected dried blood spot samples.

**Discussion:**

Migrants enrolled in the study appear to be representative of recent Filipino migrants to the U.S. Additionally, migrant and non-migrant study participants are comparable on several characteristics that we attempted to balance at baseline, including age, gender, and education. HoPES is a unique study that approximates a natural experiment from which to study the effects of immigration on obesity and other health problems. A number of innovative methodological strategies were pursued to expand the boundaries of current immigrant health research. Key to accomplishing this research was investment in building collaborative relationships with stakeholders across the U.S. and the Philippines with shared interest in the health of migrants.

## Background

Obesity rates have been rising globally and reaching epidemic proportions. [1] Since 1980, the prevalence of obesity has doubled in over 70 countries. [2] In the United States (U.S.), obesity prevalence rates were an alarming 38% among adults in 2013–2014. [3] Obesity is a serious public health issue because it is related to multiple physical health (e.g. diabetes), mental health (e.g. depression), and other problems (e.g. sleep deficiency). [4] Increasing rates of obesity and obesity-related conditions have been largely attributed to sedentary behavior and diets consisting mostly of refined carbohydrates and processed foods high in fat, salt, and sugar. [5] Other factors, such as stress, may also disrupt energy metabolism and contribute to obesity. [6, 7] There is an urgent need to study how changes in the social environment affect modifiable behaviors such as diet and physical activity worldwide. To address these risk factors for obesity, it is increasingly recognized that the environments in which people live play a critical role. This present study uses a novel study design to investigate the effects of the social environment on diet, physical activity and stress.

Immigrants are an informative population from which to study obesity. In 2013, nearly 1 of 7 persons (40 million) in the U.S. were immigrants. [8] They often show lower rates of obesity compared with their U.S.-born peers, yet these rates rise with time in the U.S. For example, the obesity prevalence rates were 8.1% among immigrants with less than one year duration in the U.S., but doubled to 16.4% among immigrants with 10–15 years, according to data from the 2003–2008 National Health Interview Survey. [9]

These rising rates of obesity with duration of stay in the U.S. are often attributed to *acculturation*, the idea that immigrants shed “traditional” behaviors and adopt “American” ones over time. [10] For example, immigrants often consume greater amounts of sugar and carbohydrates with more years in the U.S. [11] This acculturation effect may be particularly strong among those who move from low-income to high-income countries. [12] Also, immigrants may encounter stress related to adapting to new places and entry into a racially stratified society. [13, 14] Indeed, stress related to racial discrimination is related to obesity risk among ethnic minorities and immigrants. [15] Further, acculturation is not only related to observable physical changes (e.g., weight gain), but also to physiological biomarkers of stress, such as c-reactive protein, an indicator of inflammation. [16]

Although informative, the majority of studies of acculturation are often confounded due to their study designs. [17–19] A key factor is the *nutrition transition* that is occurring in rapidly developing countries. This refers to the increasing availability and consumption of processed and fatty foods, decline of nutritious foods, and decreased energy expenditures. [20] The nutrition transition raises the counterfactual of whether rates of obesity would rise over time among migrants if they had not left their home countries. Indeed, secular trends in weight gain in countries like the Philippines abound. For example, in 1980, the age-adjusted prevalence of overweight and obese among adult women in Cebu, Philippines, was 7%, but the estimates climbed to 42% by 2005. [21]

A related problem is the lack of comparison groups. The necessity of comparison groups was shown in a recent study that compared Vietnamese immigrants in New Orleans, Vietnamese returnees to Ho Chi Minh City, and Vietnamese who never left Ho Chi Minh City. [22] The use of 3 groups served as a natural experiment from which to evaluate the effect of migration on health. The authors found that immigrants had higher waist-hip ratios and body mass index (BMI) compared to the other groups. [23] This important study suggested an effect of migration on obesity independent of health selection, but still left unanswered questions due to the limitations of the cross-sectional design.

Indeed, the majority of studies of acculturation are cross-sectional; there are surprisingly few longitudinal studies of immigrant health. [17] Longitudinal studies allow for capturing life experiences (e.g. employment transitions) as they unfold, and the monitoring of changes in diet and weight gain.

One longitudinal study indicated duration was associated with higher BMI and waist circumference among Mexican immigrants, but not Chinese immigrants. [24] Another longitudinal study found that BMI rises with age among immigrant teenagers, but it was not clear if this rise can be attributable to age or duration effects. [25] Thus, the small and conflicting body of literature highlights the need for further longitudinal research.

Even more rare are studies that collect data prior to migration. Only a few examples exist. [26–28] Data collected in the country of origin before migration are essential to providing a baseline from which to evaluate change over time. This is particularly important given the effects of globalization on the wide accessibility of western foods (e.g. from chain restaurants such as McDonalds). For example, if an “American diet” is partially measured by eating fast food [29], then one would want to know how much fast food is being consumed prior to migration. Thus, in order to understand the experiences of migrants in the U.S., we need to understand their experiences prior to departure, and also to compare their experiences with persons remaining in the country of origin. [19, 30]

The Health of Philippine Emigrants Study (HoPES), reported presently, was designed to address some of these limitations and evaluate the counterfactual of whether immigrants would become obese absent migration. Three key features distinguish HoPES from most prior research: (1) it is longitudinal; (2) it involves two cohorts, one cohort that emigrates to the United States and a second cohort that remains in the Philippines; (3) it collects baseline data for both cohorts in the Philippines, and also obtains follow-up data in the US for migrants, and in the Philippines for non-migrants. HoPES also collects information regarding potential mediators (e.g. diet) and moderators (e.g. living in an ethnic enclave) of obesity risk.

Filipinos were chosen for this research for two major reasons. First, the Philippines has a strong migration stream to the U.S. In 2012, about 57,000 Filipinos became new permanent residents in the U.S. or about 1 every 10 min. [31] In 2013, the 3.5 million Filipinos in the U.S. made them the 2nd largest Asian subgroup in the country. [31, 32] Second, Filipinos show disparities in obesity-related risk factors and conditions. Filipino immigrants show higher prevalence (13.5%) of obesity compared with immigrants from India (10.3%) and China (3.3%). [33] In a study of over 1.7 million enrollees in a healthcare organization, Filipinos had the highest prevalence of diabetes among Asian subgroups: Filipinos (16.1%); South Asian (15.9%); Vietnamese (9.9%); Chinese (8.2%); and a twofold higher rate compared to Whites (7.3%). [34] Some biomarkers show similar patterns; for example hypertriglyceridemia: Filipinos (41%); Asian Indians (39%); Chinese (30%); Vietnamese (34%) . [35] These high rates provide compelling evidence to study Filipino populations further.

## Methods

### Pilot study

Our efforts began with a small pilot project designed to evaluate the feasibility of prospectively examining the health of migrants across two countries. [19] This pilot recruited 27 U.S.-bound migrants and 26 non-migrants in the Philippines. The pilot administered a 5-h assessment that included a survey, anthropometrics, a food frequency questionnaire, a 24-h food diary, and accelerometer-measured physical activity. All instruments were translated into Filipino. The pilot recruited migrants via the Pre-Departure Orientation (PDOS) sessions, described further below. An important success of the pilot was in the retention of 96% of the sample over 1 year of follow-up. This high retention is particularly notable given the challenges of following participants not only longitudinally, but overseas.

### Overall design

**Fig. 1:**
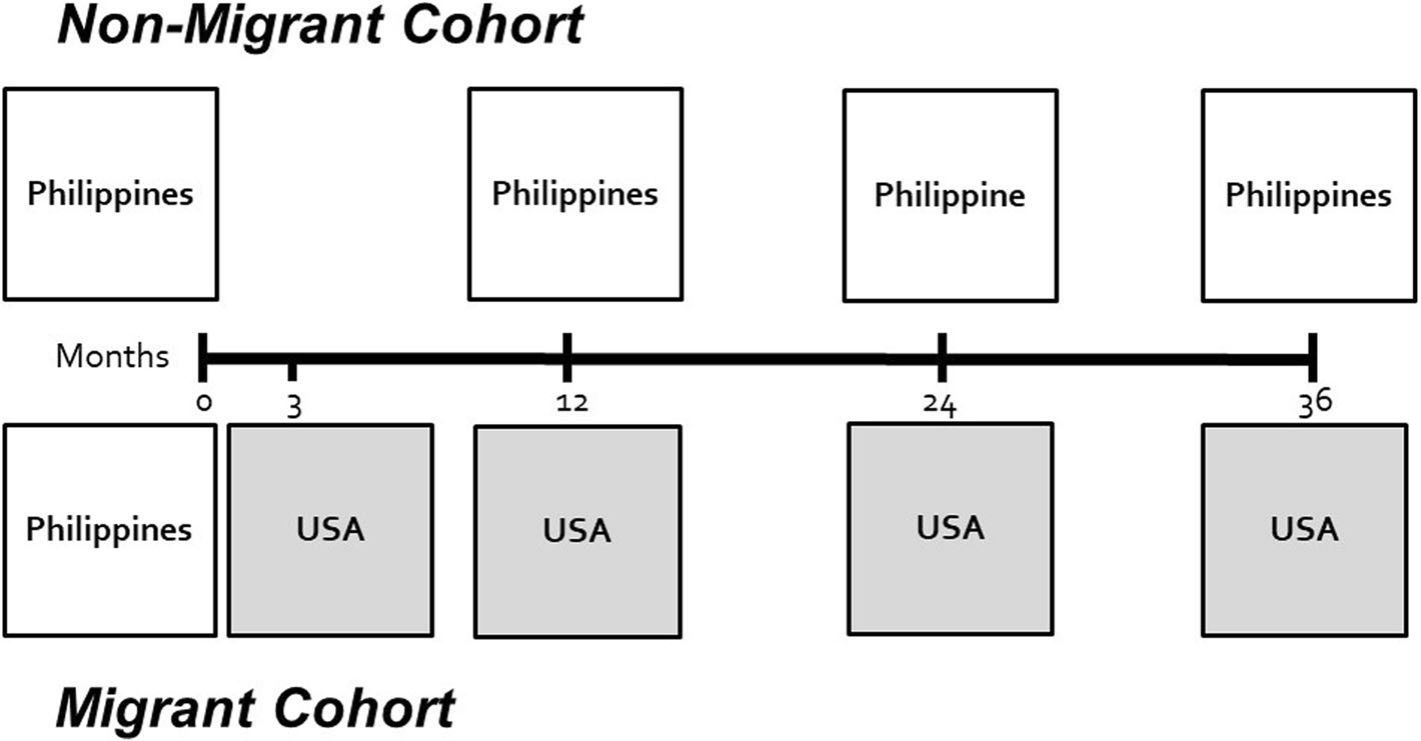
Study Design of the Health of Philippine Emigrants Study (HoPES)

The pilot study was expanded to HoPES, a representative dual-cohort, transnational, longitudinal study (Fig. 1). The first cohort consists of 832 persons who planned to emigrate from the Philippines to the United States, and the second cohort consists of 805 persons who intend to remain in the Philippines for the duration of the 3-year study. Participants were recruited from February 2017 to October, 2017, with plans to continue following the cohorts until 2020.

Baseline data for both cohorts were collected in the Philippines. Follow-up data collection for migrants will occur in the U.S. at 3-months, 12-months, 24-months, and 36-months after baseline. The 3-month assessment is designed to verify movement to the United States and to collect data regarding their immediate experiences. Non-migrants in the Philippines will be assessed on the same schedule, except they will not undergo the 3-month assessment.

### Recruitment of HoPES migrants

Migrants were recruited from the universe of legal permanent emigrants from the Philippines who were destined for the U.S. Such recruitment was made possible by a collaboration with the Commission on Filipinos Overseas (CFO), the Philippines national governmental agency that regulates emigration. After an individual has secured all of their legal permissions to migrate from the Philippines to the U.S., and received their visas, migrants are required to complete a mandatory Pre-Departure Orientation Seminar (PDOS) in the Philippines. PDOS attendees sit through a 2-h training session that provides information on their rights and obligations, advice on travel and settlement, and similar information. After attending the PDOS, attendees receive an official stamp on their passport which is verified by airport officials. Persons without this stamp are refused entry on the plane and sent to attend a PDOS.

HoPES recruited study participants from the PDOS sessions. With the permission of CFO officials, PDOS attendees were approached by HoPES staff and provided information about the study. Eligible persons were then asked to provide informed consent to join the study. HoPES staff made it explicitly clear that the decision to participate in the study does not affect a person’s legal permissions to emigrate from the Philippines to the U.S. We included persons ages 20–59 as we are interested in working-age adults. Because HoPES is a prospective study about obesity, women who were pregnant at the time of screening were not eligible for participation. Persons for whom the United States was not their final destination, who would not emigrate within the next 3 months, or who could not speak English, Tagalog, or Cebuano were also not eligible. Approximately 90% of PDOS attendees departed the country within 2 months of the session, with some departing that same day. Thus, another unique feature of HoPES is the collection of baseline data just days to weeks before attendees exit the country.

### Recruitment of non-migrants

Non-migrants were recruited via stratified random sampling of households in three strata, Metro Manila, Metro Cebu-urban and Metro Cebu-rural, which have total populations of about 12 million, 2.2 million and 400,000, respectively. Within each stratum, we created a listing of all barangays, then sampled barangay with probability proportional to population size. A *barangay* is the smallest administrative division of local government in the Philippines, similar to a U.S. census tract. We sampled 8 barangay from Manila, 7 from Cebu-urban, and 5 from Cebu-rural. Once a barangay was selected, we then obtained permission to contact residents from two gatekeepers, the Mayor of the city or municipality (who oversee several barangay), and the local barangay Captain.

Within each barangay, we obtained a cluster sample of households, and sampled individuals within households. Households were chosen by first selecting a random landmark within the barangay (e.g. a park), and then proceeding in a random direction from that landmark to sample households. For each household, we enumerated all adult residents and screened them for inclusion in the study. Eligible persons included: residence in the barangay for the past 2 years, no plans to move from the residence in the next 3 years, age 20–59, not currently pregnant, and able to speak Cebuano, Tagalog, or English. Live-in domestic workers were ineligible. One eligible person was randomly selected and invited to join the study. If they declined, we resampled within the household. Forty persons per barangay were recruited into the study.

To help ensure that the non-migrant sample was comparable to the migrant sample, we tracked the sex, age, education level and urbanicity (urban versus rural residence in the Philippines) of migrant participants recruited for this study, accumulating these frequencies in a 2x2x2x2 table (male/female × 20–34/35–59 years old x any college/no college x urban/rural). Then, non-migrants were sampled so as to recruit these target numbers in each cell. The targets were periodically readjusted to match the observed frequencies among migrants as the sample was accrued.

Table 1 shows the unweighted distributions of HoPES migrants with non-migrants for various age-gender-education strata. HoPES participants are also compared with the recent (< 2 years duration of residence) Filipino immigrants to the U.S. based on data from the 2011–13 American Community Survey. As seen in the table, the distributions are fairly comparable across the samples. For example, in the U.S., about 8% of recent immigrant Filipino immigrants are females, age 20–34 and without a college education. This same strata comprises 7% of both HoPES migrants and non-migrant cohorts.

**Table 1:** Unweighted distribution of HoPES participants at baseline vs. recent (< 2 year) Filipino immigrants in the American Community Survey (ACS), 2011–13

Age	Gender	College education	ACS (*n* = 58,287)	HoPES migrants (*n* = 832)	HoPES non-migrants (*n* = 805)
20–34	F	no	.08	.07	.07
20–34	F	yes	.25	.30	.22
20–34	M	no	.04	.04	.03
20–34	M	yes	.11	.11	.15
35–59	F	no	.11	.10	.18
35–59	F	yes	.23	.19	.21
35–59	M	no	.06	.06	.05
35–59	M	yes	.13	.12	.09

### Incentives and payments

Participants were compensated with a modest amount of cash and “load” for their cell phones (i.e. prepaid time). Snacks were available at the end of the session for the migrant participants. They were also informed about their height, weight, waist circumference, blood pressures, and cholesterol measurements. Participants with elevated values were advised to consult with a healthcare provider.

### Measures

HoPES collects a variety of data, including measured height, weight, waist and hip circumferences, lipid profile, blood pressure, and dried blood spots. It also includes a 60-min interviewer-administered questionnaire that was pre-tested prior to final administration.

#### Anthropometrics

Trained interviewers measured height, weight, and waist and hip circumference. For these measures, participants were first instructed to empty their pockets and remove shoes, heavy garments and other items that might interfere with measurement. *Waist circumference (WC)* and *hip circumference (HC)* were measured with participants standing. [36] WC is measured at the level midway between the iliac crest and the lower rib margin at the end of expiration. HC is measured as the maximum circumference over the buttocks. These measures were taken with a standardized measuring tape (Weight and Measure brand, Model CAN150). *Height* was measured using a calibrated stadiometer (Charder brand, Model HM200P). [36] *Weight* was assessed with a pre-calibrated digital scale (Tanita Corporation, Model BC-541 N), which also obtained body composition measures such as percent body fat.

Staff were trained so that inter-observer error did not differ by more than 0.5 cm for height, 0.3 kg for weight and 1 cm for girth. All measurements of height, weight, WC and HC were obtained thrice on each person by one staff person. A fourth measurement was taken if the difference between the three measurements exceed 0.5 cm for height, 0.3 kg for weight, and 1 cm for girth measurements. The mean of the 3 closest measurements was calculated for each subject.

#### Blood pressure

Systolic and diastolic blood pressure were obtained by a trained nurse in a standardized manner with an electronic blood pressure monitor (Omron Healthcare, Model BP785N). After five minutes of resting in a seated position, three consecutive measurements were obtained from the left arm (unless there was a physical health reason or injury) without restrictive clothing, while in the seated position with both feet flat on floor. Three measurements were taken and averaged.

#### Dried blood spots and lipids

A trained nurse obtained dried blood spot (DBS) samples. After cleaning with an alcohol wipe and allowing the alcohol to dry,  a fingertip (typically the ring finger) was pricked with a sterile lancet. Five blood droplets were collected through finger prick with sterile lancet. The first drop was wiped away with sterile gauze. Subsequent drops were collected onto paper collection cards (Whatman 903 Protein Saver cards). DBS cards were allowed to dry for at least 12 h at room temperature, then sealed in plastic bags with desiccant packets. DBS specimens were kept in insulated containers with ice packs, then stored in a freezer until they were shipped overnight to the Biodemography Lab at the University of Washington’s Center for Studies in Demography and Ecology for freezer storage (−80^o^ Celsius) and analysis. Assays are planned for biomarkers such as glycosylated hemoglobin (HbA1c) and C-reactive protein, with the potential for additional analyses in the future. The nurse also obtained lipids, including total cholesterol, triglycerides, and high density lipoprotein, with a point-of-care device (PST diagnostics, CardioCheck PA, CHEK-1708) following similar procedures as noted above to facilitate participant safety and reliable samples.

### Survey

Interviewers administered a survey that asked about the following domains:

#### Diet

Diet was measured with a Food Frequency Questionnaire (FFQ). An initial FFQ consisting of 157 food items was developed in the pilot study, which was modeled from the Block FFQ. [37] Items and nutrient information for Filipino foods was obtained from *The Philippine Food Composition Tables* (2010). [38] The current study used items from the pilot study’s FFQ with further input and refinement from our community partners. The efforts were led by a team of dieticians from both countries. The FFQ was supplemented with additional questions regarding how participants typically buy and prepare their food, who they usually eat with, and special diets.

#### Physical activity

Physical activity was measured with the self-reported International Physical Activity Questionnaire, which has been previously validated in the Philippines and many other countries. [39] In the pilot study, we employed accelerometers, but deemed that these devices were not feasible to use in the main study because of logistical challenges (e.g. participants were travelling and forgot to use the devices, lost them).

*Demographic factors*: age, gender, religion, marital status.

*Socioeconomic position*: education, income, occupation, remittances, financial strain, [40] food insecurity [41].

*Stress*: perceived stress, [42] unfair treatment, [43] acculturative stress [44].

*Immigration*: visa type, pre-migration planning, views of the US, reasons for immigration [45].

*Culture*: Filipino beliefs and attitudes, dietary acculturation, social identity, [46] language use and proficiency.

*Health*: self-rated health [47], depressive symptoms [48], sleep duration and disturbances [49], cognitive impairment [50], chronic health conditions [51], homesickness, recent injuries and infections, medication use, help-seeking behavior, tobacco and alcohol use [52].

*Social networks & social capital*: friends & family members in the USA and Philippines, social isolation, social capital [53].

*Geographic identifiers*: In the Philippines, we captured the barangay and province, and for non-migrants, their address. In the U.S., we assessed postal addresses. Such information will be geocoded.

*Meta-data*: time/date of data collection, interviewer’s impressions of participant’s cooperation, and presence of other persons during interview.

#### Training and quality control

HoPES had 22 staff collecting baseline data in the Philippines. These included licensed nurses, trained interviewers, team leaders, and drivers. HoPES investigators from the U.S. spent 10 days in the Philippines training staff. The interviewers underwent another 2 weeks of additional training. These trainings were designed to standardize procedures and diagnose and solve problems, and included: human subjects protections, measurement of height, weight, waist and hip circumference, dried blood spots and lipids, and survey administration. Such trainings included clarification and troubleshooting of instruments, standardization of how to collect and record the data, role playing, and practice.

To ensure quality control, the research teams had online conference meetings 2–4 times per month, weekly e-mail reports and other communications as needed. We also developed a detailed training manual that covered all study procedures.

### Statistical power

Our statistical analyses will focus on longitudinal analysis of the 4–5 assessments collected over the 3-year follow-up period. While our dependent variables include measures of adiposity (e.g., weight, BMI, hip and waist circumference), as or more important for this three-year study are measures of proximal obesity risk (i.e., dietary behavior measures, physical activity, biomarkers, stress), since changes in these may precede change in adiposity and are more likely to be detected in a study of this duration. Using the repeated measures module of Optimal Design 3.0 (http://hlmsoft.net/od/) and variability estimates from the pilot study and literature reports, we determined that a sample size of 800 *per group* at baseline (total *n* = 1600), after accounting for 20% attrition at Year 3, would provide 80% power to detect a standard effect size of 0.25 for difference in linear slope of obesity risk factors, which is considered a small effect size. Power for effect sizes of 0.2 and 0.3 are 63 and 93%, respectively. These effect sizes are within the range of those seen in the literature. For moderator analyses, power exceeds 80% for standard effect sizes of 0.35. Power for mediation analyses should also be adequate; simulation studies have shown that a sample size of 462 is adequate to achieve 80% power for product tests of small-small mediated effects when using the bias-corrected bootstrap. [54]

### Plans for follow-up and retention

Follow-up data collection at months 3, 12, and 24 will be by telephone and focus on survey data due to cost restrictions. However, follow-up at months 36 will be in-person, with plans to collect the same survey data, anthropometrics, and biomarkers measured in the baseline.

Retention is a challenge for all prospective studies, and ours is even more complicated because of the nature of migration. New migrants tend to move more than the general population. [55] Many will move within the United States and return temporarily to the Philippines. Some will change their minds about residing in the USA and permanently move back to the Philippines. Our study is designed to investigate such moves and to minimize loss to followup. We are employing various strategies, described below, that were useful in the pilot study. Although these strategies are no guarantee, they did help us achieve a 96% retention rate in the pilot study. [19] They include the following:

#### Comprehensive contact information

We ask participants to provide us with contact information including their phone numbers, e-mail addresses, mailing addresses, and social media handles. We also ask them for the contact information of family and friends in the USA and the Philippines (contact information is updated regularly).

#### Immediate “check in”

At the baseline survey, we ask for their expected dates of arrival in the United States. We contact migrants after the expected date to verify that they indeed moved and arrived in the U.S. safely.

#### 3-month survey

The main goal of the 3-month survey is to keep in touch with migrants and update their contact information. Also, as part of this survey, we send incentives to participants via postal mail. This allows us to verify their physical addresses.

#### Social media

We have an active presence on social media and regularly post news items (e.g. Filipino movies) that may be of interest to our target population. The goal of these posts is to keep participants engaged with the study. Our websites are monitored by HoPES staff to ensure confidentiality, relevance, and civility.

### Analysis

Our initial analyses will compare migrants to non-migrants on baseline characteristics. As the prospective data become available, we will compare the trajectories of several obesity risk factors between the two cohorts using longitudinal linear mixed models for continuous outcomes (a.k.a. multilevel growth models) and generalized linear mixed models for non-normal outcomes. Models will include fixed effects for age, year and group (migrant/non-migrant). The age effect models age-related changes in the dependent variable, the year effect models secular trends, and the group effect models the effect of migration. Tests for 2- and 3-way interactions among these effects correspond to tests among the 3 hypotheses of acculturation, secular trends, or both. We will also formally test for mediation for postulated mediators (e.g. stress). Finally, we will develop weights to account for the sampling design and perform analyses with these weights.

Additionally, we plan to conduct sensitivity analyses using propensity scores. [56–58] Using logistic regression with migrant/non-migrant status as the dependent variable, we will create a propensity score model and estimate propensity scores for participants. We will then match migrants with non-migrants on propensity scores within sex-age-education strata. This will yield two samples that are comparable in terms of distributions of observed covariates. We will iteratively check for covariate balance and refine the models if needed to achieve comparable groups. “Greedy” propensity score matching methods [56] are most compatible with our goals as they permit the use of most conventional multivariate procedures on the resulting samples. The matched sample can be used in analyses as is normally done using a sample created by a randomized experiment (i.e., used in analyses comparing migrants and non-migrants by including an indicator for migrant status). [56]

## Discussion

HoPES is a unique study of immigrant health featuring prospective data collection from two matched cohorts across two countries, with baseline data obtained in the country of origin. Importantly, the design approximates that of a natural experiment from which to investigate the effects of migration on health status (i.e. obesity risk). The use of a comparison cohort that does not immigrate provides a way to evaluate the counterfactual of whether health would change among immigrants had they not migrated.

Such research would provide important information from which to gauge the effects of acculturation versus global secular trends in health problems such as obesity, and advance the understanding of the needs of migrant populations with regards to the development of appropriate and effective interventions for addressing obesity and obesity-related conditions. HoPES also affords a new look into the potential effects of globalization. We expect that many migrants are already primed to quickly adopt some “western” behaviors due to the globalization of American products, ideas, and behaviors. [19] Contrary to notions that immigrants are “empty vessels” who suddenly adopt “western foods” such as hamburgers only after arrival, the data collected pre-migration data will allow us to empirically investigate the extent to which migrants already eat such foods in their home country, and to study how such “pre-acculturation” might accelerate their adoption of other western behaviors.

The HoPES study has a strong research design, but does have several limitations. First, the study’s findings may not be generalizable to immigrants of other countries, and thus, caution is warranted in extrapolating our results to other groups. Second, physical activity is based on self-report. As noted previously, our pilot study indicated that collecting objective measures of physical activity from accelerometers was not feasible due to logistical challenges. Other population-based studies have expressed similar concerns. [59, 60] While self-report measures are subject to possible reporting biases, we do use a measure that was validated in population-based studies in the Philippines and 50 other countries. [39] Third, our design is not a true experiment, which would require randomizing individuals to migration or non-migration, but nonetheless, provides a rigorous approximation to such a design. The matching of migrants and non-migrants on gender, education, and age (and sensitivity analyses with propensity score matching) provides some comparability on baseline characteristics. Fourth, the study does not include Filipinos born in the U.S. This group is not necessary to study the counterfactual of whether obesity would increase if migrants had not left the Philippines, although the addition of such a group would permit us to investigate the health of second and third generation Filipinos. To this end, other datasets are available (e.g. the National Latino and Asian American Study, Filipino American Community Psychiatric Epidemiological Study). [61, 62]

Despite these limitations, HoPES is a novel study that may contribute materially to the study of obesity. It includes novel information about pre-migration experiences, and provides an opportunity to consider the effects of acculturation alongside those of secular trends. It joins a rare group of studies that study immigrants, not only longitudinally, but also beginning in the country of origin. Such research may ultimately provide information that may be used to inform interventions and enact policies that may reduce the global burden of obesity.

## Abbreviations

DBS: Dried blood spots
FFQ: Food frequency questionnaire
HC: Hip circumference
HoPES: Health of Philippine emigrants study
PDOS: Pre-departure orientation seminar
WC: Waist circumference
